# Metachronous bilateral diabetic mastopathy mimicking breast cancer in a woman with long-standing type 2 diabetes: a case report

**DOI:** 10.3389/fmed.2026.1957813

**Published:** 2026-09-03

**Authors:** Zhixiang Kuang, Wei Ding, Li Li

**Affiliations:** Department of Ultrasound, Rizhao People’s Hospital, Rizhao, Shandong, China

**Keywords:** breast imaging, case report, core needle biopsy, diabetic mastopathy, immunohistochemistry, sclerosing lymphocytic lobulitis, type 2 diabetes mellitus

## Abstract

**Background:**

Diabetic mastopathy is an uncommon benign fibroinflammatory disorder of the breast that often resembles malignancy on clinical examination and imaging. Most reports involve younger women with long-standing type 1 diabetes. Metachronous bilateral disease in an older woman with type 2 diabetes is less often described.

**Case presentation:**

A 69-year-old woman with a 38-year history of type 2 diabetes treated with self-administered insulin had previously undergone partial excision of a suspicious right-breast mass in 2020. Histology was consistent with sclerosing lymphocytic lobulitis; immunohistochemistry demonstrated both T- and B-cell populations, a CD21-positive follicular dendritic cell network, and a low Ki-67 proliferation index. Four years later, she presented with a palpable left-breast mass. Ultrasonography showed an irregular heterogeneous hypoechoic lesion with indistinct margins and posterior acoustic shadowing, classified as BI-RADS 4b. Mammography showed an irregular mass with a lobulated contour and indistinct margins in a heterogeneously dense breast. On 3.0-T MRI, the mass was irregular and non-circumscribed, with heterogeneous internal enhancement and predominantly progressive enhancement with a slight late decline. High signal was present on diffusion-weighted imaging, but diffusion restriction could not be confirmed because the ADC map and numerical ADC value were unavailable. Core needle biopsy showed marked stromal fibrosis and periductal and perilobular lymphocytic infiltration without malignancy, concordant with diabetic mastopathy. At follow-up on 5 November 2025, the mass remained clinically and sonographically stable. No treatment was administered, and continued surveillance was advised.

**Conclusion:**

In a patient with long-standing diabetes and previous contralateral diabetic mastopathy, a new suspicious breast lesion may represent metachronous disease. Imaging alone cannot exclude cancer. In selected patients, a benign and imaging-concordant core biopsy may support avoidance of immediate repeat excision, but continued clinical and imaging surveillance remains necessary.

## Introduction

Diabetic mastopathy (DMP), also called diabetic fibrous mastopathy or sclerosing lymphocytic lobulitis, is a benign breast disorder characterized by dense stromal fibrosis and lymphocytic inflammation around lobules, ducts, and vessels. Affected patients may present with a hard mass and imaging findings that are difficult to distinguish from breast cancer (1–5).

Most reported patients are premenopausal women with long-standing type 1 diabetes and microvascular complications. DMP can also occur in older women, patients with type 2 diabetes, and men (6, 7). Lesions may be bilateral, multifocal, or recurrent, and new disease can develop after surgery (8, 9). We describe pathology-confirmed DMP in opposite breasts at an interval of approximately four years in an older woman with long-standing type 2 diabetes. The case shows how previous pathology, current imaging, and core biopsy can be combined to reach a diagnosis without another excision.

## Case presentation

### Patient information and first breast event

A 69-year-old woman had clinically diagnosed type 2 diabetes for 38 years and used self-administered insulin. The adequacy of long-term glycemic control was unknown. She also had diabetic retinopathy and nephropathy. Family history was not documented. Psychosocial history and genetic testing were not assessed. In 2020, she presented with a painless mass in the right breast. Examination found a firm, poorly mobile mass at the 10 o'clock position, approximately 2 cm from the nipple. The available clinical record stated that ultrasonography had classified the lesion as BI-RADS 4b. At surgery, it was an ill-defined firm mass measuring approximately 4.0 × 3.0 × 3.0 cm. Serum tumor-marker testing in 2020 showed CEA 2.53 ng/mL, CA19-9 37.62 U/mL, AFP 8.31 ng/mL, and CA72-4 < 1.5 U/mL. The original imaging examinations and reports from the 2020 right-breast episode, which had been performed at another hospital, were unavailable for retrospective review.

On 30 October 2020, the patient underwent partial excision and reshaping of the right breast. Histologic examination showed atrophy of ducts and lobules, focal ductal dilatation, periductal and perilobular lymphocytic infiltration with lymphoid follicle formation, extensive stromal fibrosis, and focal calcification. Immunohistochemistry showed CD3- and CD5-positive T cells, CD20- and CD79*α*-positive B cells, a CD21-positive follicular dendritic cell network, Bcl-2 and Bcl-6 positivity, CD10 negativity, and a Ki-67 proliferation index of approximately 2%. The findings supported a diagnosis of sclerosing lymphocytic lobulitis, or diabetic mastopathy. Her postoperative recovery was uncomplicated.

### Contralateral breast event

On 28 December 2024, the patient presented with a palpable mass in the left breast. On physical examination, the skin of both breasts showed no erythema or swelling. An approximately 3.0 × 2.5 cm mass was palpable in the left breast at approximately the 3 o'clock position, 3 cm from the nipple. The clinical clock-face localization was approximate; imaging localized the abnormality near the 2 o'clock position. The mass was firm, poorly defined, irregular on the surface, minimally tender, and only slightly mobile within the gland. No left axillary or supraclavicular lymphadenopathy was palpable. No mass was palpable in the right breast, and no right axillary or supraclavicular lymphadenopathy was identified. Ultrasonography demonstrated a patchy heterogeneous hypoechoic abnormality near the 2 o'clock position, measuring approximately 4.0 × 1.7 × 1.0 cm overall; a representative focal component measured 1.74 × 1.02 cm. The lesion was solid, irregular, and had non-smooth indistinct margins, heterogeneous internal echoes, posterior acoustic shadowing, and limited punctate or peripheral Doppler flow. No abnormal axillary lymph nodes were seen. These suspicious sonographic features supported classification as BI-RADS 4b.

Mammography showed heterogeneously dense breasts (breast composition category C) and an irregular mass with a lobulated contour and indistinct margins in the outer left breast, without definite calcification. Breast MRI was performed using a 3.0-T system. After intravenous administration of 12 mL of gadopentetate dimeglumine, dynamic contrast-enhanced images were acquired with a temporal resolution of 60 s per phase for seven post-contrast phases. The mass involved the upper and lower outer portions of the left breast and was irregular, with non-circumscribed margins and heterogeneous internal enhancement. The kinetic curve showed predominantly progressive enhancement with a slight late decline. The lesion showed high signal intensity on diffusion-weighted imaging; however, diffusion restriction could not be confirmed because a corresponding ADC map and numerical ADC value were unavailable. The exact first post-contrast timing, contrast injection rate and saline-flush protocol, and diffusion-weighted imaging b values were unavailable. Although long-standing diabetes and the previous contralateral diagnosis raised the possibility of DMP, the irregular/non-circumscribed morphology on ultrasonography, mammography, and MRI and the posterior acoustic shadowing on ultrasonography prevented imaging from excluding malignancy.

**Figure 1:**
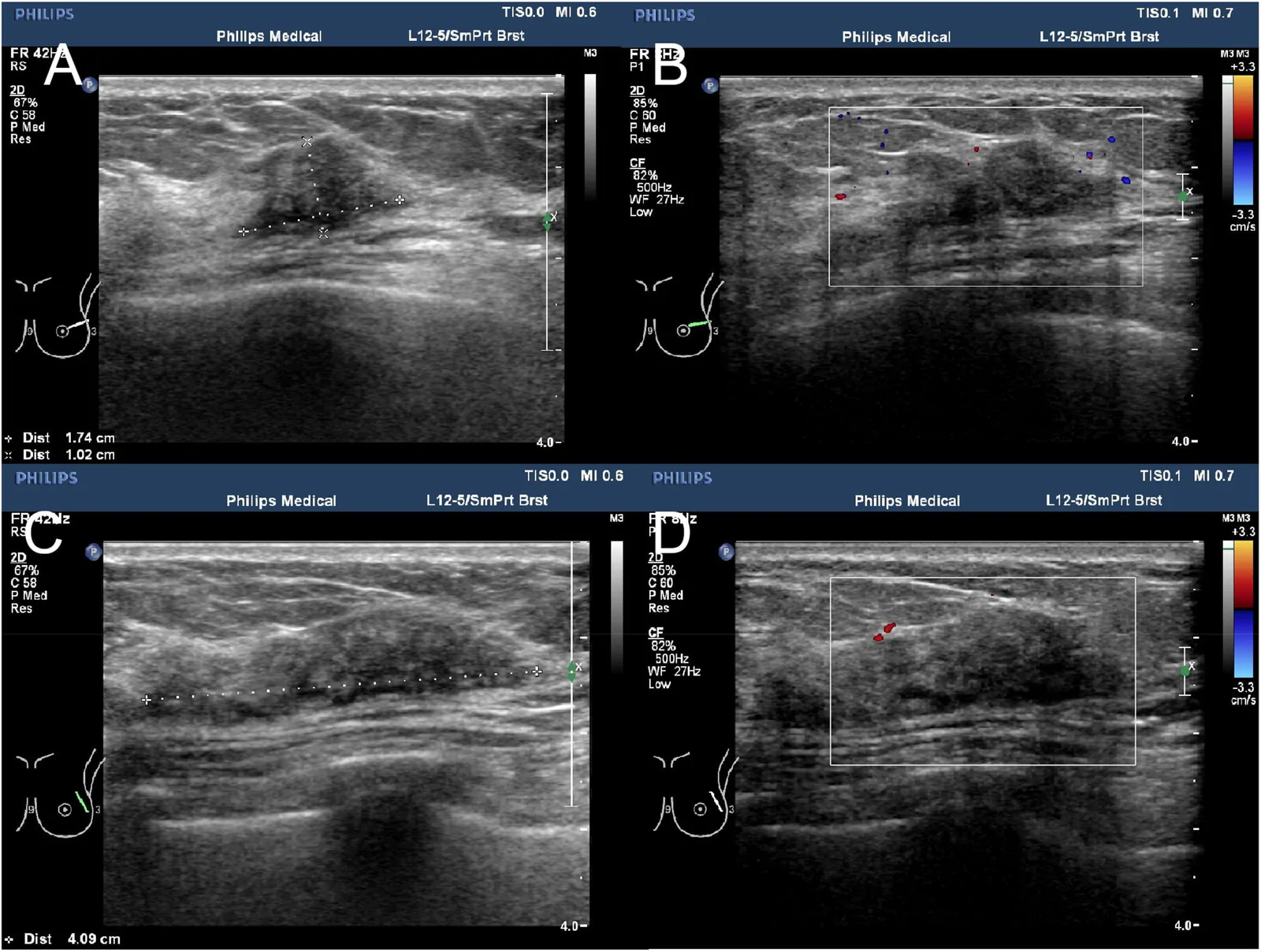
Left-breast ultrasonography in 2024. **(A)** An irregular heterogeneous hypoechoic focal component measuring approximately 1.74 × 1.02 cm. **(B)** Color Doppler imaging shows limited punctate flow. **(C)** A broader patchy hypoechoic abnormality extends approximately 4.09 cm. **(D)** Additional Doppler imaging demonstrates minimal vascularity within or around the abnormality.

**Figure 2:**
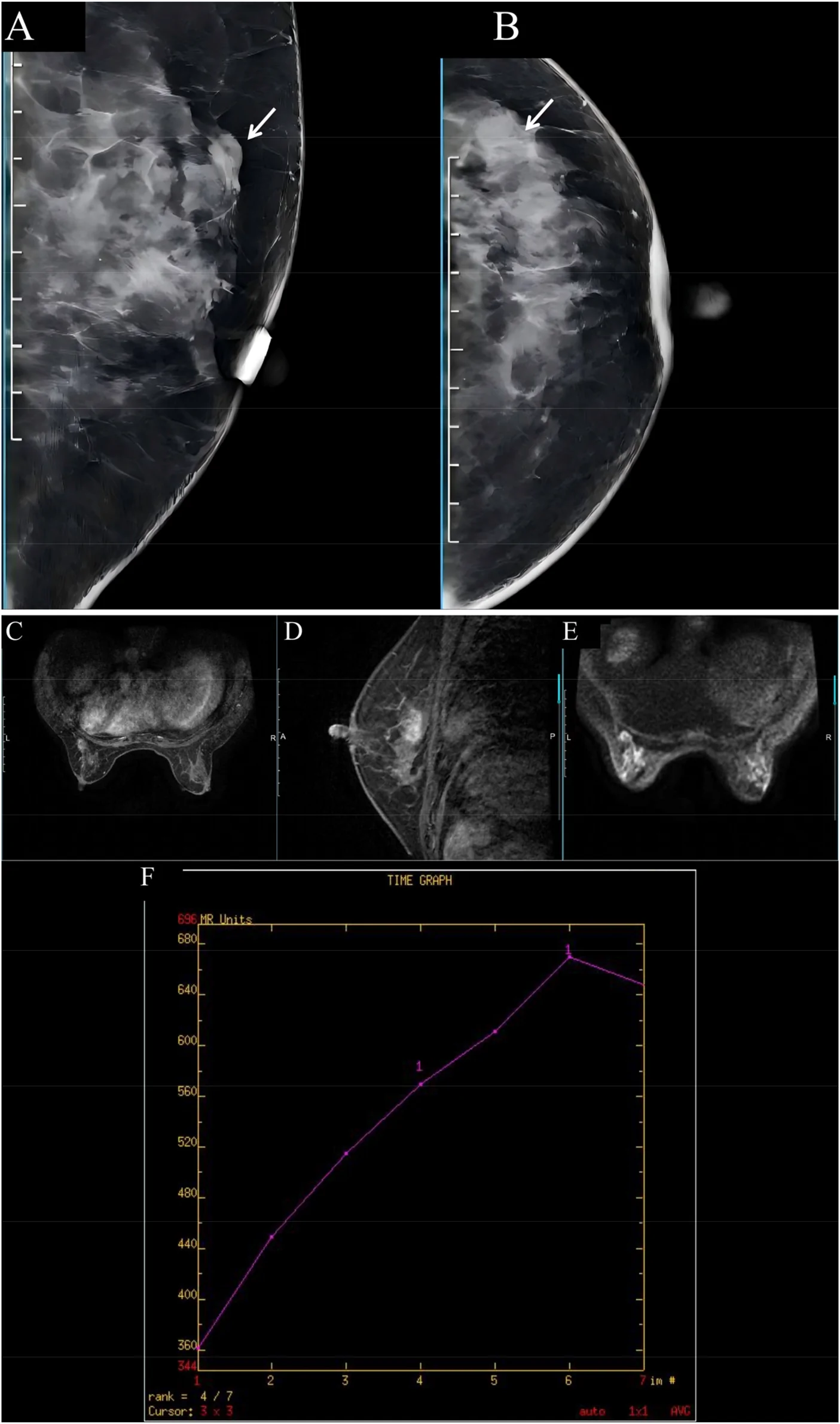
Mammographic and magnetic resonance imaging findings of the left breast in 2024. **(A,B)** Craniocaudal and mediolateral oblique mammograms show an irregular mass with a lobulated contour and indistinct margins in the outer breast (arrows), without definite calcification; the breasts are heterogeneously dense (composition category C). **(C,D)** Axial and sagittal dynamic contrast-enhanced MRI show an irregular non-circumscribed mass involving the outer breast with heterogeneous internal enhancement. **(E)** Diffusion-weighted imaging shows heterogeneous high signal; diffusion restriction could not be confirmed because the ADC map and numerical ADC value were unavailable. **(F)** The time-signal intensity curve shows predominantly progressive enhancement with a slight late decline.

**Figure 3:**
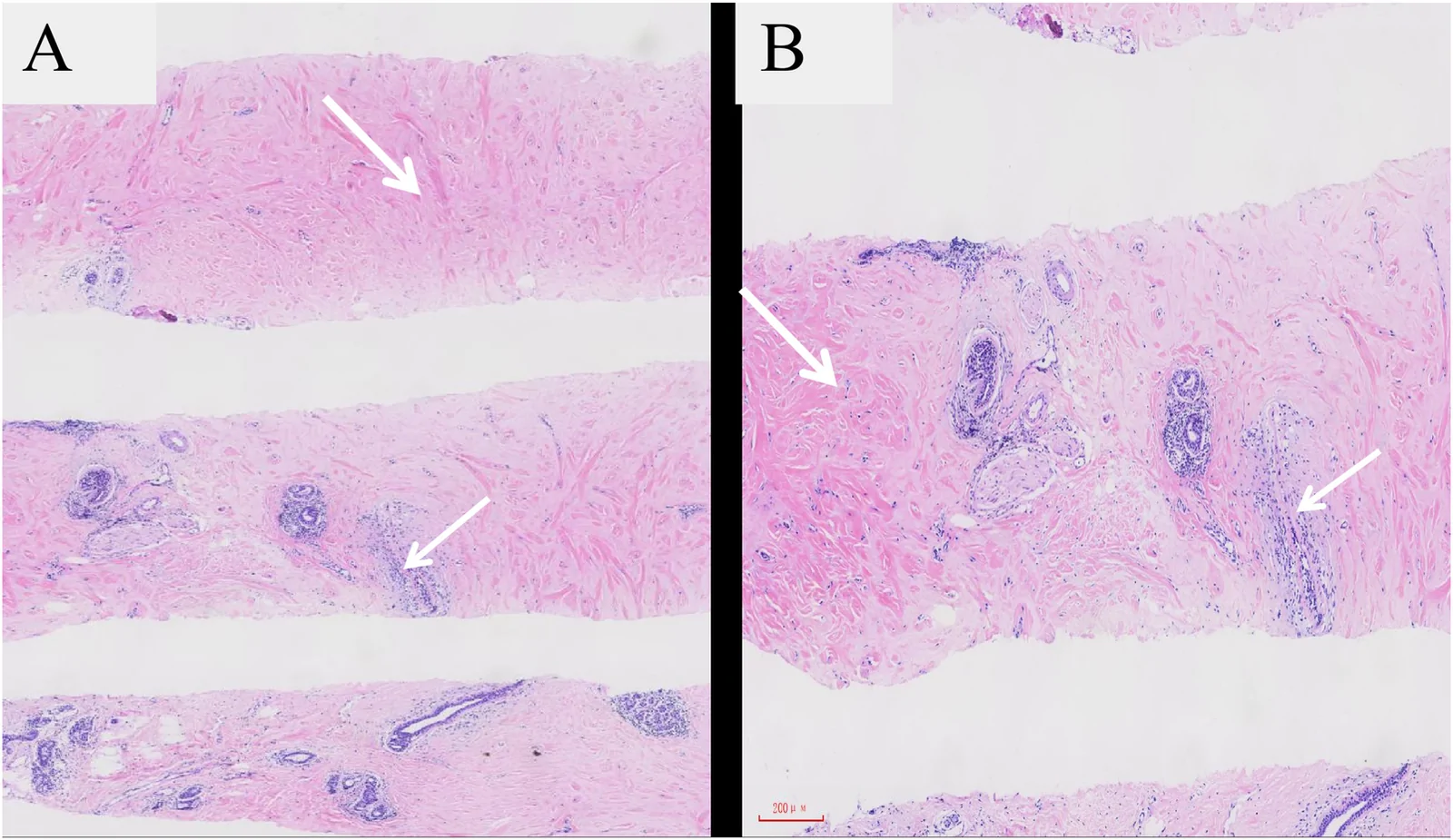
Core needle biopsy of the left outer-breast lesion (hematoxylin and eosin). Views at 20× **(A)** and 40× **(B)** magnification show marked collagenous stromal fibrosis/sclerosis with lymphocytic infiltration around ducts and lobules (arrows), supporting diabetic mastopathy in the appropriate clinical context.

On 29 December 2024, ultrasound-guided core needle biopsy of the left breast lesion showed marked stromal fibrosis and sclerosis, with lymphocytic infiltration around mammary ducts and lobules. No malignancy was identified. Immunohistochemistry was not performed on the 2024 core biopsy because the hematoxylin-and-eosin findings closely matched those of the 2020 right-breast excision, for which immunohistochemistry had supported a reactive mixed T- and B-cell infiltrate, and the current morphology, long-standing diabetes, and imaging-pathology correlation favored DMP. The pathology was therefore considered concordant with DMP after review of the clinical and imaging findings. Serum tumor-marker testing showed CEA 3.82 ng/mL, CA125 6.04 U/mL, and CA15-3 16.70 U/mL. Tests performed on the same date showed fasting glucose of 6.02 mmol/L and glycated albumin of 22.3% (clinical reference interval, 10.8%-17.1%). Creatinine was 44 μmol/L, and the estimated glomerular filtration rate was 99.88 mL/min/1.73 m^2^. The corresponding ultrasonographic, mammographic, MRI, and histopathologic findings are shown in Figures 1–3.

### Timeline

The clinical timeline is summarized in Table 1.

**Table 1:** CARE clinical timeline.

Date	Clinical and diagnostic findings	Intervention and outcome
Sep-Oct 2020	Painless right-breast mass; available records described a firm, poorly mobile lesion and BI-RADS 4b assessment. Original external imaging and reports were unavailable for retrospective review.	Surgical evaluation planned.
30 Oct 2020	Ill-defined right-breast mass, approximately 4.0 × 3.0 × 3.0 cm. Serum markers: CEA 2.53 ng/mL, CA19-9 37.62 U/mL, AFP 8.31 ng/mL, and CA72-4 < 1.5 U/mL.	Partial excision and reshaping of the right breast.
3 Nov 2020	Histology showed ductal/lobular atrophy, periductal and perilobular lymphocytic infiltration with lymphoid follicles, and extensive stromal fibrosis.	Diagnosis: sclerosing lymphocytic lobulitis/diabetic mastopathy.
28 Dec 2024	Palpable left-breast mass. Ultrasonography, mammography, and MRI showed irregular/indistinct or non-circumscribed morphology; ultrasound demonstrated posterior acoustic shadowing. BI-RADS 4b.	Malignancy could not be excluded; tissue sampling recommended.
29 Dec 2024	Core biopsy showed marked stromal fibrosis/sclerosis and periductal/perilobular lymphocytic infiltration without malignancy. No immunohistochemistry was performed. Serum markers: CEA 3.82 ng/mL, CA125 6.04 U/mL, and CA15-3 16.70 U/mL.	Imaging-pathology concordant DMP; no further surgery.
5 Nov 2025	Mass remained palpable without evident enlargement, pain, skin/nipple change, or axillary lymphadenopathy. Ultrasound showed a 4.2 × 1.6 × 1.1 cm lesion, similar to 2024. Fasting glucose 8.46 mmol/L; HbA1c 8.5%.	No treatment; continued clinical and imaging surveillance.

### Diagnostic assessment

Breast cancer was the principal diagnosis to exclude because the new solid hypoechoic lesion was irregular, had indistinct margins and posterior acoustic shadowing, and showed suspicious morphology on mammography and MRI. The differential diagnosis also included invasive lobular carcinoma, stromal fibrosis, granulomatous mastitis, lymphoma, and other lymphocytic or autoimmune breast lesions. DMP was specifically considered because of the patient's long-standing type 2 diabetes and the pathology-confirmed right-breast DMP in 2020. Nevertheless, neither the prior diagnosis, the imaging findings, nor the serum tumor-marker results could exclude a new malignancy; the marker panels also differed between 2020 and 2024 and were reported descriptively rather than used to establish the diagnosis. Ultrasound-guided core needle biopsy was selected because it preserves tissue architecture, permits assessment of stromal fibrosis and lymphocytic lobulitis, and is more reliable than fine-needle aspiration for distinguishing DMP from carcinoma. The marked stromal fibrosis/sclerosis, periductal and perilobular lymphocytic infiltration, and absence of malignancy were concordant with the imaging appearance, long-standing diabetes, and previous contralateral pathology. Subsequent stability provided supportive follow-up evidence but was not used independently to exclude cancer.

### Therapeutic intervention and follow-up

The right-breast lesion was excised in 2020. In 2024, the left-breast lesion was sampled by ultrasound-guided core needle biopsy, and no further surgery was performed. On 5 November 2025, the left-breast mass remained palpable but had not evidently enlarged. The patient reported no pain, and no skin or nipple changes or palpable axillary lymphadenopathy were identified. Follow-up ultrasonography demonstrated a 4.2 × 1.6 × 1.1 cm horizontally oriented irregular hypoechoic lesion with indistinct margins, posterior acoustic shadowing, and limited Doppler flow, similar in size to the 2024 examination. The suspicious morphology remained consistent with BI-RADS 4b; in the context of the prior imaging-pathology concordant biopsy and interval stability, DMP remained the favored diagnosis. Fasting plasma glucose was 8.46 mmol/L and hemoglobin A1c was 8.5%, indicating suboptimal glycemic control at follow-up. No treatment was administered, and continued clinical and imaging surveillance was recommended. Any new or enlarging lesion would require renewed assessment of imaging-pathology concordance and exclusion of malignancy.

### Patient perspective

In 2024, the patient accepted the recommendation for core needle biopsy. She preferred continued observation if the lesion was benign and surgical treatment if malignancy was identified. At the 2025 follow-up visit, she agreed with the recommendation to continue clinical and imaging surveillance.

## Discussion

DMP is a rare fibroinflammatory breast disorder originally described in patients with long-standing diabetes (1). DMP has been reported in older patients with type 2 diabetes, although younger, usually premenopausal women with type 1 diabetes remain the more typical population (5–7). Histologic findings typically include dense, sometimes keloid-like stromal collagen, lymphocytic lobulitis and ductitis, perivascular lymphocytic inflammation, and variable epithelioid fibroblasts (2, 3). Its pathogenesis remains uncertain and is probably multifactorial. Proposed mechanisms include chronic hyperglycemia-related nonenzymatic glycation and advanced glycation end-product accumulation, with increased collagen production, reduced collagen degradation, and cytokine-mediated fibrosis, together with autoimmune responses involving breast ductal and stromal tissues (1, 2, 5). In insulin-treated patients, inflammatory responses to exogenous insulin and cross-reactive anti-insulin antibodies have also been proposed as contributors to ductal inflammation (5, 11, 12). Lifelong insulin exposure and autoimmune susceptibility may help explain the predominance in type 1 diabetes. In type 2 diabetes, long disease duration, eventual insulin dependence, insulin resistance, and prolonged glycemic injury may contribute, but mechanisms specific to postmenopausal patients are not established; accordingly, DMP remains uncommon in this population (5–7).

In this patient, DMP developed in the opposite breast approximately four years after excision of the right-sided lesion. The left-sided lesion was confirmed by core needle biopsy. Because it arose in the contralateral breast, it is better described as metachronous bilateral DMP than as a local recurrence. The 2020 immunophenotype—CD3- and CD5-positive T cells, CD20- and CD79*α*-positive B cells, a CD21-positive follicular dendritic cell network, Bcl-2 and Bcl-6 positivity, CD10 negativity, and a Ki-67 proliferation index of approximately 2%—supported a reactive lymphoid infiltrate in the characteristic fibrosclerotic background. Immunohistochemistry was not repeated on the 2024 core because its hematoxylin-and-eosin appearance closely reproduced the prior lesion and showed no malignancy. This reasoning supports imaging-pathology concordance, but it does not imply that immunohistochemistry can be omitted when morphology is atypical or lymphoma remains a concern.

The imaging appearances of DMP and breast cancer overlap. On ultrasonography, DMP often appears as an irregular hypoechoic lesion with indistinct or spiculated margins, variable posterior shadowing, and little or no vascularity. Mammography may be normal or may show focal asymmetry or an irregular noncalcified mass with indistinct margins. MRI findings vary; gradual progressive enhancement may favor a benign lesion, whereas irregular morphology and high diffusion-weighted signal can still resemble cancer (4, 5, 10). In this patient, ultrasonographic posterior acoustic shadowing resulted from attenuation of the sound beam by the densely fibrotic tissue but is also a suspicious feature encountered in carcinoma. The prior diagnosis of DMP therefore did not remove the need for biopsy because the new lesion remained suspicious across ultrasonography, mammography, and MRI.

Core needle biopsy is usually more informative than fine-needle aspiration in suspected DMP because dense fibrosis may yield scant cytologic material, whereas core samples preserve tissue architecture for evaluation of stromal sclerosis and periductal and perilobular inflammation (4, 5). In selected patients, a benign and imaging-concordant core biopsy may support avoidance of immediate repeat excision. This approach is relevant because DMP can be multifocal or bilateral and can recur or appear after excision. In a cohort of 28 patients, 55% underwent surgery, 32% had postoperative recurrence, and coincident breast cancer was also reported (9). A previous diagnosis of DMP should therefore not be used to dismiss a new suspicious mass; each new lesion requires appropriate tissue assessment, imaging-pathology correlation, and surveillance.

This report is limited by gaps in the available clinical record. The original images and reports from the 2020 right-breast episode were unavailable for retrospective review. The adequacy of long-term glycemic control before 2025 and the duration of retinopathy and nephropathy were unknown. Family history was not documented, and psychosocial history and genetic testing were not assessed. Laboratory-specific reference intervals were not available for the retrospectively retrieved tumor-marker results, so the values are reported without classification as normal or abnormal. Immunohistochemistry was not performed on the 2024 left-breast core; although the hematoxylin-and-eosin findings were typical and concordant with the 2020 pathology and current imaging, the two lesions therefore cannot be compared immunophenotypically. For the 2024 MRI, a numerical ADC value and ADC map, the exact first post-contrast timing, the injection rate and saline-flush protocol, and diffusion-weighted imaging b values were unavailable. Accordingly, diffusion restriction could not be confirmed and no standardized formal kinetic-curve category was assigned. Finally, only one follow-up evaluation, approximately 10 months after biopsy, was available; therefore, longer-term stability and the long-term safety of surveillance cannot yet be established.

## Conclusion

A new suspicious breast lesion in a patient with long-standing diabetes and previous contralateral sclerosing lymphocytic lobulitis may represent metachronous bilateral DMP. Because imaging cannot reliably distinguish DMP from cancer, tissue diagnosis remains necessary. In selected patients, a benign and imaging-concordant core biopsy may support avoidance of immediate repeat excision. Continued clinical and imaging surveillance remains necessary.

## Data Availability

De-identified data supporting this case report may be requested from the corresponding author, subject to institutional approval and patient privacy requirements.
